# Maternal nutritional supplement delivery in developing countries: a scoping review

**DOI:** 10.1186/s40795-019-0270-2

**Published:** 2019-02-11

**Authors:** L. McKerricher, P. Petrucka

**Affiliations:** University of Saskatchewan, Saskatoon, S7N 5C9 Canada

**Keywords:** Maternal nutrition, Nutritional supplement, Developing countries, Low-income countries

## Abstract

**Background:**

Maternal under-nutrition in low-income countries has been inextricably linked to negative child outcomes. Developing countries lack policies for monitoring and evaluating maternal nutritional programs, which has led to a gap in data collection regarding the effectiveness of prenatal supplement delivery methods. The objective of this scoping review is to examine and determine the delivery methods of maternal nutritional supplements in developing countries.

**Methods:**

Scoping review of maternal supplement programs delivery methods in low-income countries including Bangladesh, Ethiopia, India, Kenya, and Nepal are examined. A systematic search was performed in six databases; CINAHL (Cumulative Index to Nursing & Allied Health), MEDLINE, Web of Science, PubMed, Scopus, and FSTA (Food Science and Technology Abstracts).

**Results:**

A systematic search performed in six databases yielded a total of 510 un-duplicated results; (CINAHL: 42, Medline: 112, Web of Science: 77, PubMed: 46, Scopus: 179, FSTA: 38, and additional records: 16). Results after duplicates were removed (*n* = 308), these results were screened, and relevant studies based on the research question identified and selected (*n* = 12). The 12 full-text articles were assessed for eligibility and 8 of these studies were excluded for not meeting the scoping review criteria. Data was extracted and charted from the four remaining studies. The findings were collated and summarized.

Three modes of delivery were identified: 1. Volunteer maternal nutrition educator delivered supplements to the pregnant woman’s home; 2. The pregnant woman received a maternal supplements from school, health/local center, or village market; and 3. The pregnant woman received a ration card for subsidized food.

**Conclusions:**

Barriers in delivering maternal supplements included lack of trained professional volunteers, limited support and guidance provided to volunteers, and a high cost of equipment, supplies, and buildings. Pregnant women in developing countries faced many obstacles in accessing maternal supplement programs including poverty, rural isolation, limited transportation, low social status, traditional, cultural, and religious practices. Strategies required to improve program delivery involved an earlier invitation to prenatal supplements, increase in partnerships, a focus on adolescent girls’ health, paid maternal leave, increase in training and incentives for volunteers, and self-help groups focused on prenatal education and counseling services.

## Background

Maternal undernutrition contributed to one-third of child deaths under the age of five [1]. Prenatal nutrition is critical to fetal growth, healthy development, and survival of future generations [2]. Many developing countries realize the importance of maternal nutrition and have developed programs to aid in improving prenatal health and education. Interventions by UNICEF and most national governments have implemented are promoting breastfeeding and complementary feeding, providing vitamin A, iron, and folic acid supplementation, supporting universal salt iodization, promoting global food fortification, and supporting home fortification programs. UNICEF (2009) identified three effective nutrition interventions: maternal nutrition during pregnancy and lactation; breastfeeding (first hour of birth to 24months of age); and adequate complementary feeding and micronutrient intervention (6 months and onward). Despite this progress, UNICEF (2009) reported that approximately 195 million children under the age of five in developing countries suffered from chronic nutritional deprivation, a consequence of maternal malnourishment. International commitments and efforts to ensure adequate maternal nutrition and to reduce child mortality are evident, but there continues to be a lack of policy development to guide maternal nutrition program interventions and to ensure adequate monitoring and evaluation of program delivery methods [3]. This scoping review examined the existing literature and identified a gap in the evaluation of maternal nutrition supplement delivery methods. A conceptual analysis is required to interpret issues surrounding delivery methods to further inform future research and decision-making.

Evidence has shown that poverty, poor education, social inequalities, rural isolation, culture, and religion play a crucial role in the complexity of maternal nutritional supplement program delivery methods [4, 5]. Investing in strategies to improve nutritional interventions in low-income countries will be influential to the health and well-being of future generations as indicated in the sustainability development goals. This paper examines and identifies the delivery methods utilized by maternal nutritional supplement programs in developing countries, explores barriers and suggests strategies for improvement.

## Methods

### Search strategy

A priori inclusion and exclusion criteria were developed by both reviewers to pre-defined the objectives and methods for the scoping review. An initial systematic limited search was performed in a selection of relevant databases to find research-based articles on delivery methods for maternal nutritional supplements in developing countries. A text word analysis to search each database including various key terms, mesh terms, and subject headings; maternal nutrition, prenatal nutrition, antenatal nutrition, pregnant, pregnancy, vitamin, supplement, AND/OR diet, AND third world country/countries, developing country/countries, AND/OR low-income country/countries, AND delivery, access, programs, agencies, provision, AND/OR distribution. A second search, using all the terms identified above, was undertaken in six relevant databases; CINAHL (Cumulative Index to Nursing & Allied Health), MEDLINE, Web of Science, PubMed, Scopus, and FSTA (Food Science and Technology Abstracts). A reference management software, Mendeley™, was used for recording and organizing all relevant bibliographic citations for the scoping review. The reference lists of all identified articles were searched for additional studies. A systematic search performed in six databases yielded a total of 510 un-duplicated results; (CINAHL: 42, Medline: 112, Web of Science: 77, PubMed: 46, Scopus: 179, FSTA: 38, additional records from other sources: 16). Results after duplicates were removed (*n* = 308).

### Relevance screen and inclusion criteria

**Fig. 1:**
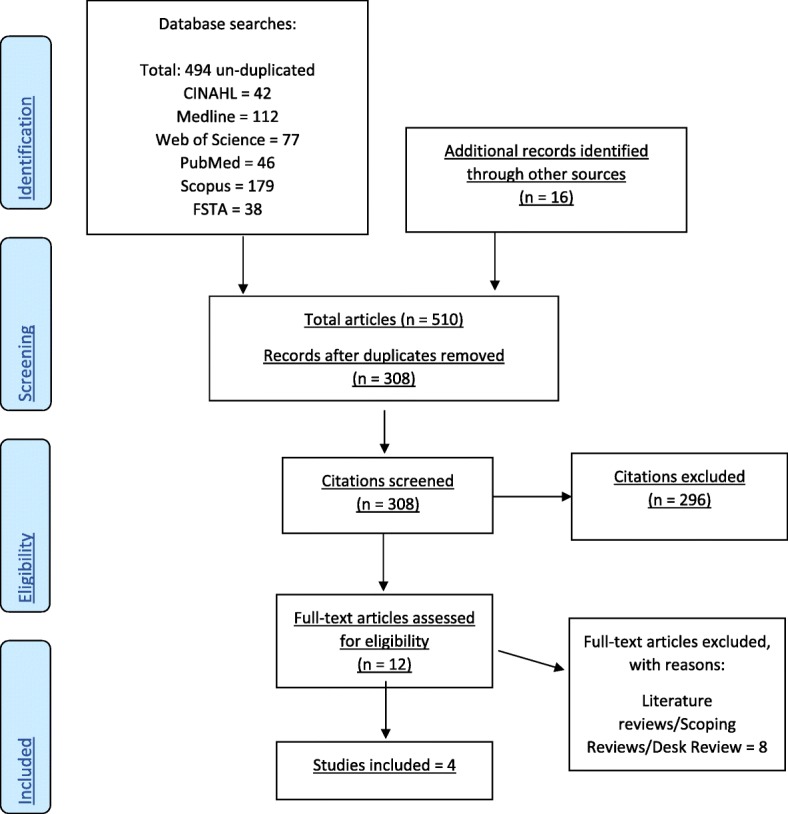
PRISMA Flow Diagram of articles through the scoping review process updated May 22, 2018. 10.1371/journal.pmed1000097

Inclusion screening criteria was developed by the reviewers for the scoping review. The results (*n* = 308) were screened based on the inclusion criteria. The initial inclusion screening criteria included relevant articles published after January 1, 2011. Only English language articles were considered as this was an initial review and unfunded research, hence our efforts were to achieve the broadest manageable data set. Peer-reviewed articles were considered relevant if they addressed the research objective. Only research-based full-text articles were included in the scoping review. The second screen was performed by both reviewers, where articles were extracted based on the title and abstract relevance. From this screening process, twelve articles met the inclusion criteria and were assessed for eligibility. Both reviewers read the twelve full-text articles and determined that eight of these studies were not research-based. These eight studies were excluded for being literature reviews, scoping reviews, or desk reviews. Therefore, four of the articles were included in the scoping review (see Fig. 1).

### Study selection

Four full-texted research-based articles that answered the research objective were selected and main characteristics including study methodology, sample population, location, delivery methods interventions, outcomes, key findings, and recommendations were extracted.

### Scoping review management, data charting, and analysis

Data was collected, analyzed, and synthesized into a Microsoft Word document using a pinch chart to facilitate categorization and organization. The reviewers identified diverse interventions and, although the interventions tried to achieve the same results, they differed in nature, providing clinical heterogeneity. All articles were peer reviewed, listed in and accessible through one or more of the selected databases, and included ethics and/or necessary administrative approvals.

## Results

### Delivery methods

In the scoping review, there was an abundance of journal articles that addressed maternal supplements but few that focused on how to deliver the supplements. The scoping review of maternal supplement programs in developing contexts (which, in this case, included Bangladesh, Ethiopia, India, Kenya, and Nepal), identified three delivery methods for distributing maternal supplements to women during the prenatal period. The first mode reflected programs which had volunteer maternal nutrition educators deliver supplements to the pregnant women’s homes. The second was pregnant women received maternal supplements from school, health center/local center, or village markets. In the final instance, the pregnant women received a ration card to purchase subsidized food.

#### Volunteer maternal nutrition educator delivers supplements

In the volunteer maternal nutrition educator delivered approach, maternal supplements and/or food rations were brought to the pregnant women’s home. Workers distributed Iron-Folic Acid (IFA) supplements house-to-house in distant communities [6]. This delivery method allowed the pregnant woman to receive the supplements without the inconvenience of traveling during pregnancy.

#### Pregnant woman receives supplements

In two studies, from Bangladesh, pregnant women received and consumed food supplements from a local community nutrition center distributed by a volunteer [7, 8]. In both studies, the volunteers were local women trained by the implementing government organization to provide supplement packages with nutrition education [7]. The package contained rice, lentils, molasses, oil, and vegetable protein, and was meant to supplement daily food intake already being consumed [7]. Both Bangladesh programs were sponsored by the government.

#### Subsidized food or cash transfer

Subsidized food was provided to low-income households, pregnant women received a ration card to purchase subsidized food. In India, the Targeted Public Distribution System subsidized food, mostly rice, pulses, and sugars, to below poverty line households [5]. “According to the Targeted Public Distribution System approximately 64% (12,200,000) of all households possessed a Below Poverty Line ration card as of August 2010” [5].

In Ethiopia, the Productive Safety Net Program provided cash transfers or food supports to households whose adults participated in labor-intensive public works projects [6]. The program offered financial compensation to exempt pregnant and lactating women from public work for six months after their child’s birth [6]. The program offers an increased calorie intake per household but does not address the food quality [6]. To be eligible for this program pregnant women are required to participate in four antenatal care visits including IFA supplementation to receive a cash transfer or food support [6].

### Barriers for programs

#### Sustainability

From the reviewed literature, it is apparent that sustainability of programs relies on government policies, human resources, communication networks including transportation, and fragile health system infrastructures [9]. The main issue is the low level of awareness among policymakers regarding the severity and consequences that maternal undernourishment has on the population which may, in turn, contribute to the low prioritization of maternal nutrition program management [5].

#### Cost, resources, and social status

Poverty is a major barrier which limits access to essential health services. Maternal nutrition is heavily affected by social phenomena including poverty, caste discrimination, and the low social status of women. Pregnant women are unable to afford to purchase the food recommended by nutritional programs [6]. Insufficient food in the household was a major reason for poor diet as well as the sharing of prenatal supplements and food rations with the rest of the household because their family members were also malnourished [5, 6].

Most maternal nutrition programs struggle with limited resources, resulting in shortages of buildings, personnel, equipment, supplies (vitamins, contraceptives, and food), and professional training/support [5]. Limited support and guidance are given to those who are responsible for delivering nutrition services. Volunteer healthcare workers reported having heavy workloads and poor transportation which prevented them from providing adequate education and services to many pregnant women [6]. High turnover rates among voluntary community health workers lead to poor leadership, lack of experience, and difficulty in forming relationships with program participants [6].

#### Barriers for participants

There is a lack of awareness and education among pregnant women who participated in nutrition programs. For example, some pregnant women viewed IFA as anemia treatment rather than prevention. In Ethiopia, a key barrier to the nutritional program was that participants lacked awareness of government guidelines for IFA during pregnancy [6].

Traditional beliefs and customs also affected supplement consumption among pregnant women. Some pregnant women believed “eating down”, defined as eating small amounts, would reduce the babies’ weight, and if they ate too much there would be less space for the fetus to grow in their stomach [6]. Volunteer healthcare workers suggested eating small amounts frequently, but pregnant women could not eat multiple small meals throughout the day because it is considered taboo to eat alone [6]. Religious fasting and taking laxatives was also believed to reduce the babies’ weight and allow for an easier birth [6]. There was also a strong religious belief that everything is in God’s hands including nutrition and health [6].

Earlier access to nutrition programs resulted in a higher total nutrient intake for mother and baby [7]. The earlier in pregnancy the food program was implemented resulted in better maternal-infant interactions and improved fetal growth and development [7]. Mothers, who were not offered the maternal supplements, suffered from food insecurity resulting in infant distress, high levels of personal stress, and/or depression, leading to poor maternal-infant interactions [7].

Access also relied on the timely delivery of food rations. Some nutrition program participants reported that it took 1–2 months for food rations to arrive and distribution sites were 3–30 h from their communities making it extremely difficult to obtain especially later in their pregnancy [6]. Some programs refused women as eligible to receive food supplements because of their lack of attendance for antenatal care education. Non-eligible participants felt food distribution was unfair and families who needed food rations were not always considered eligible [6].

Pregnant participants were counseled to rest during their pregnancy; however, this was not possible because of the enormous work burdens including household chores, collecting water, and agricultural duties [6]. In Sub-Saharan Africa, 7.6 million people were eligible for the Productive Safety Net Program which provided $4 US or 15 kg of unfortified grain per month and exempted participants from work for 6 months during lactation [6]. However, no data was available on how many women were registered and participated in this incentive program [6].

#### Strategies to improve program delivery modes

Noznesky et al., (2012) suggested delivering newlywed packages of IFA supplements/ nutritional supplements to all young women who are at risk for anemia before they become pregnant. There needs to be a system for identifying and delivering supplements to all malnourished pregnant women to ensure full coverage of services. Household mapping that is regularly updated could help with identifying the target population [5]. The system should also include data on who received supplements/food rations and monitor the number of antenatal educational/health care visits [5]. Technology can be used to develop and implement a data management system for nutritional programs [5].

Incentives for trained professionals could be offered to work in remote and rural areas and build public-private partnerships to coordinate implementing nutritional interventions [5]. Manageable workloads should be constructed for volunteers that offer support and guidance through supervision and telecommunications. Volunteers should emphasize teaching lifelong skills, such as home gardening, cooking, and nutritional education, utilizing the food that is available to the participants [5]. Volunteers should educate and support community members to introduce their own initiatives that are appropriate for local culture, tradition, and religious beliefs [5].

Success is based on the program’s ability to improve nutritional status and education of pregnant women, build partnerships, and improve coordination [6, 9]. Government partnerships will elevate the priority to develop policies and strengthen program interventions [5]. Offering benefits, such as cash transfers to exempt pregnant and lactating women from public work, would enable the mother to purchase food and develop maternal-infant relationships. Improving the monitoring and evaluation system of maternal nutritional programs is essential to measure the effectiveness of delivery methods.

## Discussion

The three modes of delivering maternal supplements in developing countries revealed in this review were through: 1. Volunteer maternal nutrition educator delivered supplements to pregnant woman’s home; 2. The pregnant woman received maternal supplements from school, health center/local center, or village market; and 3. The pregnant woman received ration card for subsidized food. Each of these modes was examined and it was identified that there is a lack of documenting and monitoring within the nutritional programs which contributed to the uncertainty of success and efficiencies. For example, the IFA supplements workers distributed house-house but did not document or monitor which women received supplements [6]. In India, the Targeted Public Distribution System, lacked data collection on which individuals purchased food with their ration card or if the food was consumed [5]. Data collection and monitoring is vital to improving nutritional program delivery. Through data collection, programs can begin to develop and optimize evidence-based delivery interventions and evaluate the outcomes of these modalities to address maternal nutrition.

## Recommendations for further studies

The effectiveness of maternal supplements is well recognized in the literature; however, future research should investigate effective delivery strategies. Delivery strategies need to scale up coverage for the targeted population and reduce disparities by providing equitable access [9]. A successful delivery platform should reach a large amount of the targeted population providing essential nutrition services and have equitable coverage. Upon entering the vicious circle, an infant malnourished from birth through childhood inevitably is stunted or malnourished and becomes a malnourished adult who will likely reproduce the next generation of stunted and malnourished offspring [1]. There needs to be a broad focus on improving adolescent girls and women health in order to break intergenerational cycles of malnutrition [1]. Malnourished adolescent girls are starting families; delivery methods need to focus on effective approaches for nutrition interventions for this specific population [9].

Numerous programs currently have nutritional interventions for improving maternal nutrition, however there is a shortage of qualified personnel for delivery services [10]. Further research should look at how to recruit and retain staff for delivery services and/or options for outsourcing delivery services through a private partnership [10]. Programs may need to invest in transportation services that would expand coverage and increase access for the targeted population.

In order to improve delivery, participants’ utilization of maternal supplements and food rations must increase. “Respondents often differentiated between what they were told to do and what they actually did” [6, p. 41–42). Programs need to consider the participants’ perceptions, culture, traditions, religious beliefs, personal priorities, and barriers for compliance to maternal nutrition program interventions. Without examining social norm perceptions neither programs nor participants will reap benefits. Until these factors are explored, programs cannot effectively evaluate and address delivery methods. Improving demand through education, self-efficacy, and social norm perceptions has the potential to achieve high maternal nutrition participation [10].

A significant limitation to this study is the use of English only evidence which favours the dominant Western science and perspectives. A future review would benefit from consideration of non-English studies.

## Conclusions

A child’s development is affected by the mother’s nutrition before and during pregnancy. Maternal malnutrition causes a generational cycle of undernutrition leading to poor health outcomes. Barriers of maternal nutrition are multifaceted including food insecurity, poverty, social norms, discrimination, traditional, cultural, and religious beliefs. Nutritional programs need to focus on providing early prenatal food supplements, expanding coverage of delivery, providing cash incentives for pregnancy leave from work, focusing on adolescent health, increasing training and support for volunteers, offering incentives to retain valuable volunteers, and developing government policymakers’ partnerships to set clear guidelines for program implementation. Maternal nutritional programs face multiple barriers to effectively deliver supplements and education to pregnant women. In this scoping review, three delivery methods were identified but results are inconclusive in examining program delivery methods because of a lack of data in monitoring and evaluating nutritional interventions.

## Abbreviations

CINAHL: Cumulative Index to Nursing and Allied Health
FSTA: Food Science and Technology Abstracts
IFA: Iron Folic Acid
UNICEF: United Nations International Children’s Emergency Fund
